# Chemical and Pharmacological Aspects of Capsaicin

**DOI:** 10.3390/molecules16021253

**Published:** 2011-01-28

**Authors:** Maria de Lourdes Reyes-Escogido, Edith G. Gonzalez-Mondragon, Erika Vazquez-Tzompantzi

**Affiliations:** Centro de Investigación y Asistencia en Tecnología y Diseño del Estado de Jalisco, A.C., Unidad Sureste, Calle 30 No. 151 por 7 y 7A, García Gineres, Mérida, 97070 Yucatán, Mexico

**Keywords:** capsaicin, capsinoids, synthesis, pharmacology

## Abstract

Capsaicin is a unique alkaloid found primarily in the fruit of the *Capsicum* genus and is what provides its spicy flavor. Generally extracted directly from fruit, high demand has driven the use of established methods to increase production through extraction and characterization. Over time these methods have improved, usually be applying existing techniques in conjunction. An increasingly wide range of potential applications has increased interest in capsaicin. Especially compelling are the promising results of medical studies showing possible beneficial effects in many diseases. Capsaicin’s pungency has limited its use in clinical trials to support its biological activity. Characterization and extraction/ synthesis of non-pungent analogues is in progress. A review is made of capsaicin research focusing mainly on its production, synthesis, characterization and pharmacology, including some of its main potential clinical uses in humans.

## 1. Introduction

**Figure 1:**
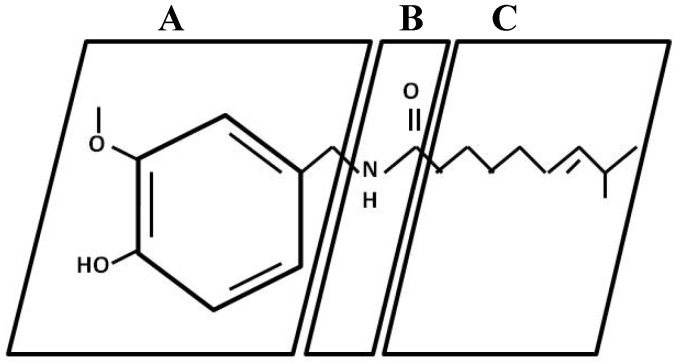
Regions of the molecule of capsaicin. A (aromatic ring); B (amide bond); and C (hydrophobic side chain).

Compounds known as capsaicinoids cause the spicy flavor (pungency) of chili pepper fruit. The primary capsaicinoid in chili pepper is capsaicin, followed by dihydrocapsaicin, nordihydrocapsaicin, homodihydrocapsaicin and homocapsaicin. Capsaicin and dihydrocapsaicin account for approximately 90% of capsaicinoids in chili pepper fruit, are the two most potent capsaicinoids and their molecules differ only in the saturation of the acyl group [1,2,3]. Capsaicin (trans-8-methyl-*N*-vanillyl-6-nonenamide) is a crystalline, lipophilic, colorless and odorless alkaloid with the molecular formula C_18_H_27_NO_3_. Its molecular weight is 305.40 g/mol, and it is fat-, alcohol- and oil-soluble. First crystallized in 1876 by Tresh, who named it, capsaicin’s molecular structure was resolved by Nelson and Dawson in 1919 [4]. Capsaicin displays *cis/trans* isomerism because the double bond prevents internal rotation. Capsaicin is always found as the *trans* isomer because in the *cis* form, the -CH(CH_3_)_2_ and the longer chain on the other side of the double bond will be close together, causing them to repel each other slightly; this steric hindrance does not exist in the *trans* isomer. This additional strain imposed causes the *cis* isomer to be a less stable arrangement than the *trans* isomer (Figure 1) [5].

Structure-activity relationships (SAR) for capsaicin agonists (a substance that is capable of binding to a receptor and elicit a response in the cell), have previously been rationalized by dividing the capsaicin molecule into three regions: A (aromatic ring), B (amide bond) and C (hydrophobic side chain) (Figure 1). It is known that the substituents in the positions 3 and 4 of the A-ring are essential for potent agonist activity, and the phenolic 4-OH group in capsaicin analogues is of particular importance, H-bond donor/acceptor properties of the phenol group are key for agonist activity [6].C-Region SAR in capsaicin analogues have been discussed in detail in some reports. In summary, a hydrophobic group, e.g., an octyl chain or substituted benzyl or group, is required for high potency. Optimally, such aralkyl groups are substituted in the *para* position by small hydrophobic moieties. [2], by other way Barbero *et al*., [7] reported the importance of lateral chain lengths for the bioactivity of capsaicinoids, which was higher between 8 and 9 carbons atoms. 

**Figure 2:**
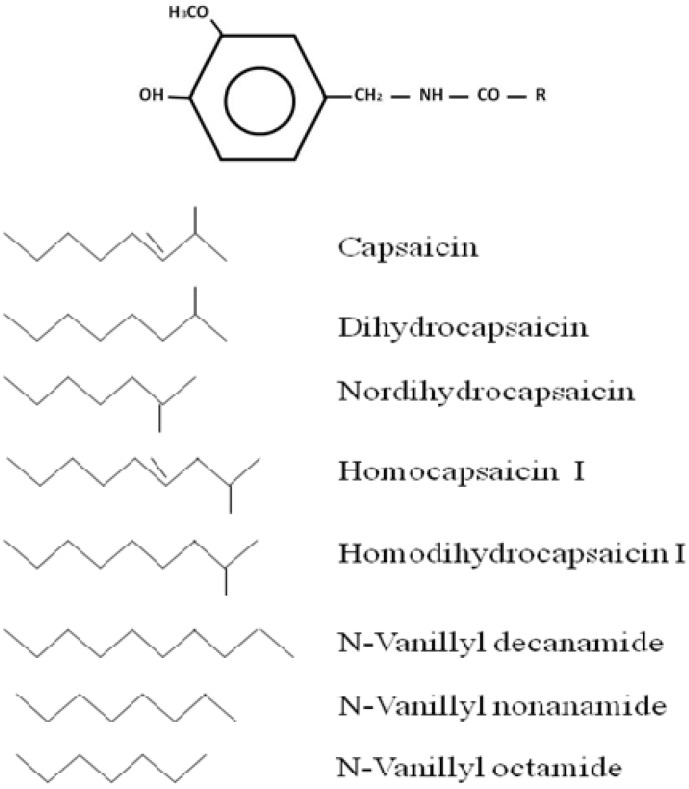
Chemical structure of different capsaicinoids and their analogues. R Capsaicinoids and analogues.

The more than twenty known capsaicinoids are all amides formed from condensation of vanillylamine and fatty acids of different chain lengths. The forms of different natural capsaicinoids depend on the number of lateral chain carbons (R) or the presence or absence of unsaturations (Figure 2). Capsaicinoids are synthesized naturally in the placenta of chili fruits from enzymatic condensation of vanillylamine and different-sized fatty acid chains which are elongated by a fatty acid synthase. This condensation is caused by the capsaicin synthase enzyme (CS) acting specifically on fatty acid chain length, which requires Mg^2+^, ATP and coenzyme A (CoA) and vanillylamine, the phenolic portion, formed from phenylalanine as a product in the phenylpropanoid pathway, while the fatty acid is formed from the amino acids valine or leucine. Structural differences among the capsaicinoids are defined by the nature of the lateral chain, which ranges from 9 to 11 carbons long with a variable number of double bonds located in different positions along the chain (Figure 2).

Capsaicinoids are important in the food and pharmaceutical industries. For this reason, a number of researchers are engaged in improving their production be it by manipulating chili plant cultivation conditions [8], chemical synthesis [9], enzymatic synthesis [10,11] or alternative methods such as cell or tissue culture [12]. To date, research has shown that capsaicinoids, and capsaicin in particular, have a wide variety of biological and physiological activities which provide them functions such as antioxidants [13], anticarcinogenics [14], promotion of energy metabolism and suppression of fat accumulation [15], and anti-inflammatories [16]. However, the potential applications of these molecules are limited by the irritation caused by their pungency; this has driven the search for and characterization of analogous molecules without inherent and undesirable effects [14,17,18].

## 2. Capsaicin Production and Synthesis

### 2.1. Genetic Regulation of Capsaicin Synthesis

Given the economic and agricultural importance of capsaicin, surprisingly little data exists on the genetics of this compound’s biosynthesis pathway. It is known that chili genotypes exhibit wide variation in capsaicin accumulation in response to genetic and environmental factors [19]. The first genetic analysis of capsaicin accumulation was done by molecular mapping and revealed the presence of a Quantitative Trait Locus (QTL) called *cap* that may contribute to increasing pungency level [20]. Most of the isolated genes related to capsaicinoids are involved in their biosynthesis, and little is known about the location and action of specific genes controlling capsaicin accumulation. Transcript accumulation has been reported for the genes *Pal, Ca4h* and *Comt*, corresponding to the phenylpropanoid pathway [21], and the genes *Kas*, *Acl* and *Fat*, which code for the enzymes involved in fatty acid metabolism [22]. Spiciness is known to be inherited as a dominant trait and is associated with the *Pun1* locus [23]. The product of *Pun1* is AT3, corresponding to a putative acyltransferase, and specific to the placenta in pungent genotypes. Its highest expression coincides with capsaicinoid accumulation, meaning *Pun1* exhibits a dominant behavior which determines pungent genotype occurrence [24]. Initially, it was thought that AT3 could correspond to CS. However, the *csy1* gene was isolated and found to code for CS [25], and its sequence is different from that of AT3, indicating that *Pun1* does not codify for CS. It was eventually established that the product of *Pun1* is related to development of the vesicles where capsaicinoids accumulate and not to their production, although formation of these vesicles is vital for pungent phenotype occurrence [26].

### 2.2. Capsaicin Biosynthesis in Plants

Capsaicin biosynthesis in plants is defined by two pathways: phenylpropanoid, which determines phenolic structure; and fatty acid metabolism, which determines the molecule’s fatty acids [27]. Capsaicin concentration increases gradually during fruit development reaching maximum levels at 40 to 50 days [28], after which it tends to degrade into secondary compounds due to peroxidase action [29]. Research aimed at increasing or improving pungent compound production has revealed that hydric stress increases capsaicinoid levels because water deficit affects the phenylpropanoid pathway [30]. Hydric stress also increases capsaicin levels by raising activity of the enzymes phenylalanine ammonia-lyase (PAL), cinnamic acid-4-hydroxylase (C4H) and CS, all involved in capsaicin biosynthesis [8]. Administration of the capsaicin precursors 8-methyl-noneic acid and vanillylamine had demonstrated that 8-methylnoneic acid occurs at lower levels than vanillylamine and is therefore the limiting substrate for capsaicin synthesis [31]. These results suggest the possibility of controlling capsaicin synthesis in the plant by manipulating substrate concentrations and water availability, which would be a cost-effective, viable alternative for increasing capsaicin production.

### 2.3. Chemical and Enzymatic Synthesis

Interest in capsaicinoids has increased in response to their multiple properties, mainly those with potential medical applications [32]. However, clinical application of capsaicin is limited because of it pungent properties. In response, greater research effort has been applied to synthesizing natural capsaicinoids as well as synthetics with properties analogous to natural types [17].

Enzyme-catalyzed synthesis has advantages over chemical synthesis in that it can be done using non-toxic reagents and substrate specificity [33]. Kobata and colleagues did a number of studies involving synthesis of capsaicin analogues using amidation reactions catalyzed with different lipases [34]. In these trials, vanillylamine condensed with fatty acid derivatives, used as a substrate in the oleose phase, resulted in 40-59% capsaicin yields and synthesis of various analogues at 2–44% yields and with from 4–18 carbons [35]. This same research group used natural oils as substrates in different reaction media (e.g. n-hexane) to synthesized non-pungent analogues [34].

**Figure 3:**
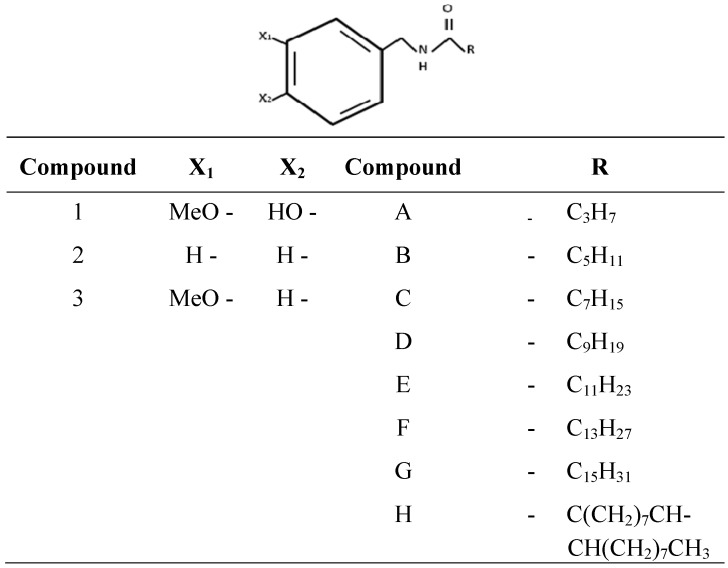
Synthesis of capsaicin analogues using different amines and donors as substrates.

Castillo and colleagues synthesized non-pungent analogues using different acyl chain lengths and chemical substitutes in the aromatic ring. They obtained two pungent analogues and other very low pungency analogues with potential uses (Figure 3) [10,11].

Capsaicin and its analogs are produced industrially using chlorinated fatty acids and amines at temperatures between 140 and 170 °C under moderate pressure [36]. A number of studies have been done using chemical synthesis of capsaicinoids. Choi and Yoon [37] used bioisosterism to synthesized 1-hydroxy-2-pyridone, a capsaicin analogue with similar biological effects. A study using chemoselective esterification of phenolic acids with alcohols for vanillyl nonanoate synthesis involved reactions between vanillyl alcohol and nonanoic acid using tetrahydrofurane as reaction medium and equimolar amounts of diisopropyl azodicarboxylate and triphenyl phosphine. Run at room temperature for 24 h, the reaction produced a 67% vanillyl nonanoate yield [38]. In another study, cerium chlorate (III) was used as a reaction catalyst in selective esterification of phenolic alcohols, resulting in a 70% vanillyl nonanoate yield [39]. Research has also been done on combined chemical-enzymatic synthesis of the non-pungent analogue dihydronorcapsaicin β-D-glucopyranoside [40]. The limited success of chemical synthesis of capsaicin is due to the toxicity of the required reagents, a disadvantage which makes enzymatic synthesis an appealing alternative to traditional chemical synthesis. 

**Figure 4:**
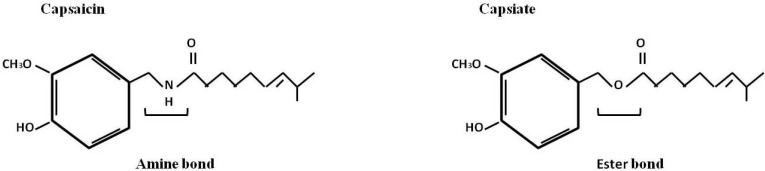
Structures of capsaicin and capsiate. Capsiate has an ester bond, whereas capsaicin has an amide bond at the equivalent position.

Capsinoids other analogues of capsaicin were recently identified from the non pungent type of red pepper CH-19 sweet, being the principal capsinoids the capsiate, dihydrocapsiate and nordihydrocapsiate [41]. Capsinoids has a remarkable structural resemblance to capsaicinoids except for their center linkage: an amide moiety in capsaicinoid and an ester moiety in capsinoid (Figure 4). Studies have shown that bio-potency of capsinoids is similar to capsaicin without the pungency or sensory irritation. Although the interest of some groups has been focused in these molecules [17,42], until now their mechanisms of action are poorly understood. These compounds are found naturally in peppers, but only in a few varieties. To obtain a sufficient volume of these capsaicinoids they will need to be synthesized, however the presence of the terminal methyl group and double bond complicates their synthesis and increases the cost [7].

### 2.4. In Vitro Synthesis

A promising alternative for synthesis of capsaicin and its analogues is *in vitro* synthesis. Capsaicinoid production via cell or tissue culture can be augmented be addition of biosynthetic pathway precursors and intermediaries as phenylalanine, ferulic acid and vanillylamine showing encouraging results [43]. Cell suspensions were employed to study the influence of phenylalanine and other phenylpropanoids, in this study the addition of 100 µM of either phenylalanine, cinnamic acid or caffeic acids did not cause significant increment in capsaicinoids content during the growth cycle. But the addition of 100 µM of vanillin, vanillylamine, *p*-coumaric acid and ferulic acid did increase 10, 7.5, 5.2 and 2.5 fold higher the capsaicin production as compared to control, respectively [44]. Other compounds such as coumaric acid and elicitors such as phycocyanin, synapinic acid, salicylic acid and curdlan result in different capsaicin production levels [45]. Accumulation of capsaicinoids and the intermediary vanillylamine have also been reported with use of the inducers salicylic acid and methyl jasmonate in cell suspension cultures [46]. Likewise, administration of the supplements L-ascorbic acid and D-limonene in the culture medium produced an approximately three-fold increase in capsaicin production [47]. A 45% increase in capsaicin production has been reported using callus cultures resistant to *p*-fluorophenylalanine [48]. Addition of the polyamine putrescine to suspension cell cultures has been shown to increase capsaicin production and activity of capsaicin synthase, the capsaicin biosynthesis terminal enzyme [49].

*In vitro* capsaicin production has been more efficient in immobilized cell cultures than in cell suspension cultures because the former use precursors for capsaicin synthesis whereas the latter use them for primary metabolism. This was demonstrated in a comparative study of both culture types using radioactively-marked phenylalanine and cinnamic acid to monitor their incorporation into capsaicin and/or its precursors. Incorporation of both compounds was higher in the immobilized cell cultures than in the suspension cultures because in the suspension cultures the phenylalanine and cinnamic acid were incorporated into protein metabolism and the cell wall, resulting in lower capsaicin production [50]. The same phenomenon has been reported for placental tissue cultures, with higher capsaicin production in immobilized versus suspension cultures; this is to be expected to some degree since the placenta contains the biochemical machinery needed for capsaicin synthesis [51]. In other study using cultures derived from placenta of *Capsicum annuum* L. which were tried with 100% nitrogen stress increased capsaicin level by 9.8 fold of that in control after 15 days of subculturing. Addition of 4 mM tyrosine to 100% phosphorus and 100% potassium stress led to 8.4 fold and 7.5 fold higher capsaicin production as compared with the control, respectively. They used various precursors too; 4 mM coumaric acid, 5 mM ferulic acid, and 4 mM vanillin which showed 6.3, 4.7 and 2.4 fold increases, respectively, being coumaric acid the most effective. Finally, they used the elicitors phycocyanin (0.25%), sinapic acid (0.05 mM), salicylic acid (2 mM) and curdlan (0.0625%), being the sinapic acid the most effective which increased capsaicin up to 8.9 fold in the cell suspension cultures. Although salicylic acid and curdlan had not effect, curdlan in combination with tyrosine, increased the accumulation of capsaicin 8.7 fold, and had an effect in the phenylalanine ammonialyase activity [45].

Putative capsaicin precursors and analogues isolated from *Capsicum* placenta and cell suspension cultures transform products into capsaicin glycoside and other identified compounds. It is also possible that peroxidases from *Capsicum* fruit and cell suspension cultures produce capsaicinoids. *In vitro* culture is clearly a promising alternative for capsaicin production since it allows for control of culture conditions and is scalable.

## 3. Capsaicin Characterization

Growing interest in capsaicin has led to its characterization with methods such as spectrophotometry UV-VIS and chromatography. These have been modified over time to develop more sensitive, faster capsaicin characterization techniques.

Thin-layer chromatography has been used to detect capsaicin on ground pepper [52], and multi-band thin-layer chromatography has been used to evaluate the R_f_ of capsaicin in different adsorbents and the same mobile phase under identical conditions (*i.e.* time, temperature, solvent vapor equilibrium, *etc.*) [53]. High-performance liquid chromatography (HPLC) has been widely used for characterization of capsaicin and it analogues [54,55,56,57,58,59]. Using different detectors, such as fluorescence, HPLC has been used to study capsaicinoids [60]. For example, this method has been applied to detect capsaicin, zucapsaicin and civamide (a capsaicin *cis*-isomer) within a 1 to 100 ng/mL concentration interval [61], and to characterize six capsaicin analogues up to a 3 ng concentration in animal tissues and blood [62]. This method was also used to quantify air-borne capsaicin and dihydrocapsaicin at a pickling and pepper manufacturing plant. Capsaicin concentration was 0.015 and dihydrocapsaicin concentration was 0.02 μg per sample; the capsaicin:dihydrocapsaicin ratio remained within a 0.3:1 to 0.5:1 range, not coinciding with the higher capsaicin content in *Capsicum* [63]. Storage stability in capsaicin solutions has been monitored using HPLC during a one year period, in a concentration range of 0.5 to 128 µM and four temperature/light combinations: 4 °C in darkness; room temperature in darkness; room temperature in light; and −20 °C in darkness. Under the tested conditions, the 4 µM treatment exhibited optimum performance, remaining stable for one year when stored at 4 °C in darkness [64]. In another study, HPLC with a diode array detector allowed quantification of capsaicin at levels below 100 Scoville units (the Scoville unit indicates the amount of capsaicin present) when mixed with nordihydrocapsaicin and dihydrocapsaicin, and at levels below 1000 Scoville units when mixed with spices such as cumin and pepper [65]. Reverse-phase HPLC was applied to identify and quantify four lipidic hydroperoxide isomers produced by oxidation of linoleic acid to evaluate capsaicin’s antioxidant activity [66].

Technological advances in the use of HPLC for organic compound analysis have been applied to capsaicin characterization, specifically liquid chromatography-mass spectrometry (LC-MS) [67,68,69] and liquid chromatography-tandem mass spectrometry (LC-MS-MS) [70]. The latter method was used to analyze capsaicinoid content in commercial pepper sprays with a standard quantification curve within a 10 to 1,000 ng/mL concentration interval. Capsaicin, dihydrocapsaicin and nonivamide were identified in the sprays within a 0.7 to 40.5 mg/mL interval [71]. In other applications, LC-MS-MS has been used to quantify capsaicin, dihydrocapsaicin and nonivamide in the blood and tissues of rats exposed to inhalations of these three compounds with resulting capsaicin concentrations of 1.0 to 90.4 ng/mL in blood, 5.0 to 167 pg/mg in lungs, and 2.0 to 3.4 pg/mg in liver [72]. Simultaneous quantification of capsaicin and dihydrocapsaicin in extracts from different *Capsicum* genotypes has been accomplished using liquid chromatography-electrospray/time-of-flight mass spectrometry with detection limits of 6 ng for capsaicin and 1.2 ng for dihydrocapsaicin [73,74]. Liquid chromatography quadrupole ion trap mass spectrometry (LC-ESI/MS/MS) has been successfully applied to quantify capsaicin’s *in vitro* half-life (2.3 to 4.1 min) [75], as well as blood capsaicin content in rats subcutaneously administered capsaicin and dihydrocapsaicin [76].

In a same way gas chromatography (GC) has been used in the study of capsaicin [77]. In a comparison of capsaicin detection using GC and HPLC, the detection limit was 1.0 µg/g with a linear capsaicin calibration curve of 1 to 250 µg/mL with GC and 0.5 to 50 µg/mL with HPLC [78]. The two methods have also been used in tandem (GCL) for determining the pungency in raw spices, extracts, food and pharmaceutical products. The pungency data obtained by both methods, the sensory test and CGL, ranged from 50,000 to 2,000,000 Scoville units with a correlation coefficient of 0.95 by a period of one year [79,80].

Solid phase microextraction-gas chromatography-mass spectrometry is another method used to study capsaicinoids [81]. In an analysis of eleven pepper varieties and four pepper-based sauces, use of this method did not require derivatization, which is required with GC, and it effectively identified and quantified capsaicin (55–25,459 µg/g) and dihydrocapsaicin (93–1,130 µg/g) in the peppers (4.3–717.3 µg/g) and sauces (1.0–134.8 µg/g) [82].

A modification of capillary chromatography that has been applied to capsaicin and dihydrocapsaicin in different varieties of *Capsicum frutescens*, is the micellar electrokinetic capillary chromatography. In this method, samples are separated by differential partition between micelles (pseudo stationary stage) and the mobile phase (aqueous buffer at pH 9). Both capsaicin and dihydrocapsaicin were detected within 11 min with an excellent resolution [83].

Capsaicin’s hydrophobic nature suggests that it may influence membrane structure, which was studied using the dipalmitoylphosphatidylcholine and dielaidoylphosphatidylethanolamine membrane models and the differential scanning calorimetry, fluorescent probe spectroscopy and ^31^P-nuclear magnetic resonance techniques. The results were used to propose the possible implications of capsaicin’s effects in biological membranes [84].

Proton nuclear magnetic resonance (^1^H-NMR) and carbon nuclear magnetic resonance (^13^C-NMR) were used in a comparative analysis of capsaicinoids purity and structure [85]. ^1^H-NMR has also been used in quantitative analysis of capsaicin in *Capsicum frutescens L.* without previous derivatization [86]. ^13^C-NMR, ^1^H-NMR and α- and β-glucosydase hydrolysis were used to identify the structure of two capsaicin glucosides: capsaicin-β-D-glucopyranoside and dihydrocapsaicin-β-D-glucopyranoside. In this study, the glucosides were not detected in non-pungent *C. annum* crops and a positive correlation was observed between capsaicin and dihydrocapsaicin levels and their glucosides [87].

The UV-VIS spectrophotometric method is one of the most inexpensive and accessible for capsaicin quantification; indeed, most laboratories have a UV-VIS spectrophotometer. However, analysis is restricted to capsaicin solutions with microgram-level concentrations [88].

Kachoosangi *et al.* [89] discuss capsaicin detection methods other than chromatography and spectroscopy with detection ranges from 0.5 to 35 µM, while capsaicin’s molecular and electronic structure can be analyzed using the density functional theory [90].

## 4. Capsaicin Pharmacology

### 4.1. Pharmacokinetics

Capsaicin is known to be effectively absorbed topically from the skin. In a study of 12 subjects topically administered a 3% capsaicin solution in three different vehicles (70% isopropyl alcohol; mineral oil; and propylene glycol in 20% alcohol), capsaicin was shown to be rapidly absorbed and to quickly reach maximum concentration when capsaicin is applied topically. The half-life of capsaicin was approximately 24 h [91].

In an extensive study of capsaicin distribution in tissues, elimination and other active principles in animals with oral administration, nearly 94% of orally administered capsaicin was absorbed and maximum concentration in the blood was reached 1 h after administration. In addition, a maximum distribution of 24.4% of administered capsaicin in blood, liver, kidney and intestine was seen in 1h and then diminished notably until being undetected after 4 days [92].

Capsaicin metabolism is apparently similar in human, rat and dog microsome. Three major metabolites have been identified for capsaicin: 16-hydroxycapsaicin, 17-hydroxycapsaicin and 16,17-dihydrocapsaicin [93]. *In vitro* studies in human skin, capsaicin biotransformation was found to be slow and most capsaicin remained unchanged while a small fraction was metabolized to vanillylamine and vanillyl acid. This suggests that cytochrome P_450_ enzymes participate minimally in capsaicin transformation in skin in comparison to their role in hepatic metabolism. Capsaicin is mainly eliminated by the kidneys with a small untransformed proportion excreted in the feces and urine [92,94].

### 4.2. Mechanism of Action

From the past decade the mechanism of action of capsaicin has been extensively studied. Capsaicin can bond to transient receptor potential vanilloid 1 (TRPV1), previously known as the vanilloid receptor, which is mainly expressed in the sensory neurons [95]. First identified and cloned in rats, TRPV1 contains 838 amino acids and has a molecular weight of 95 kDa in both humans and rats, consisting of six transmembrane domains with a short pore-forming region between the fifth and sixth transmembrane domains [96]. This receptor is a non-selective, ligand-operated cationic channel located primarily in the small fibers of nociceptive neurons. TRPV1 is also broadly distributed in tissues of the brain, bladder, kidneys, intestines, keratinocytes of epidermis, glial cells, liver, and polymorphonuclear granulocytes, mast cells, and macrophages [95,97]. It couples with a non-specific cation channel permeable to sodium and calcium ions, and is located in the plasma membrane and the endoplasmic reticulum where regulates intracellular calcium levels [98,99]. This channel can be regulated and activated by endogenous substance as endovanilloids and diverse exogenous stimuli including chemical agonists as capsaicin, olvanil and resiniferatoxin, ligands highly lipophilic that share structural similarity to several endogenous fatty acids identified as TRPV1 agonists [100]. Someone compounds are being used as antagonists for TRPV1, including capsazepine, iodo-resiniferatoxin, ruthenium red, A-425619, SB-366791, AMG9810, and SB-705498 [101]. The TRPV1 contains a heat-sensitive subunit responsible for the burning sensation caused by capsaicin. Bonding of capsaicin to TRPV1 increases intracellular calcium, triggering release of neuropeptides such as substance P and the calcium gene-related peptide (CGRP). Contact between capsaicin and sensory neurons produces pain, inflammation and a localized heat sensation. When applied locally to skin, it promotes an analgesic response due to desensitizing of the sensory neurons caused by substance P depletion [102]. This mechanism has served as a base for studies of the structure-activity relationship which are aimed at development of new synthetic ligands for the TRPV1. Capsaicin’s effects in the nervous system are not exclusively analgesic. A number of studies include results showing that it participates in release of somatostatine, CGRP and endotheline [103,104,105,106,107].

### 4.3. Clinical Applications in Humans

Capsaicin has been attributed pharmacological effects since ancient times, but not until the past twenty years has extensive research been done to determine specific applications, including in the gastrointestinal tract, for weight-loss and as an analgesic.

Research into the possible protective role of capsaicin in the gastrointestinal tract have produced controversial results and clearly indicates the need for further clinical trials to better define effective dosages. At low concentrations (0.13–160 μM) in rats, capsaicin is reported to protect the gastric mucous against ulceration by ethanol [108], whereas when administered at high concentrations (1 or 2 mg/mL) in rat stomachs it is reported to worsen damage to the gastric mucous caused by ethanol or aspirin [109]. In a study involving 84 healthy human subjects, capsaicin was found to have protective properties against ethanol- and indomethacine-associated gastropathy, with a dose-dependent decrease in base gastric acid output (ED_50_ for 400 μg capsaicin) and increased gastric emptying [110]. A possible explanation is that capsaicin mediates the antiulcerous effect by vasodilation and increasing gastric mucous blood flow (GMBF) and this is mediated by nitric oxide and CGRP release by these TRPV1 bearing cells [110].

Nearly 20 years ago, another major pharmacological effect of capsaicin was identified when it was found that it reduced adipose tissue in rodents by enhancing energy and lipid metabolism, possibly by increasing catecholamine secretion from the adrenal medulla in response to activation of the sympathetic nervous system [111,112]. These findings have helped to support further research into capsaicin’s possible therapeutic effects and mechanisms in obesity treatment.

As mentioned above, capsaicin has been included in topical treatments aimed at relief of different neuropathic pain conditions, although it can produce skin irritation. Capsaicin and its analogues have been used in topical creams and patches to treat chronic pain syndromes such as post-herpetic neuralgia, musculoskeletal pain, diabetic neuropathy, osteoarthritis and rheumatoid arthritis [113,114]. It has also been applied to treat pain from rashes, psoriasis, mastectomy, and bladder disorders. Adverse effects (burning, stinging and erythema) are normally limited to the application site, although respiratory irritations from cream inhalation and occasional systemic effects have been reported [115]. Its actual efficacy in pain relief is still somewhat in doubt due to the small number of participants and diverse definitions of pain used in studies. However, it is apparently a good alternative for pain relief in patients who do not respond to other therapies, be it when administered alone or together with other treatments [116].

## 5. Conclusions

Advances in analytical techniques are been used in the study capsaicin to increase the knowledge about its molecular characterization and structural-activity relationship. Low production yield has led to development of new synthesis strategies, including improving its biosynthesis in the plant by manipulating growth conditions or adding supplements. Other options are chemical and enzymatic synthesis or *in vitro* cell culture, all of which are promising for production of capsaicin and its analogues because they allow control of reactions to generate molecules with desired characteristics. Capsaicin is an important molecule in areas such as medicine but its pungency limits use in clinical trials. Non-pungent analog molecules have been developed in response. This is a promising molecule with many possible clinical applications but the paucity of clinical trials prevents its widespread pharmacological use.
